# Construction of face databases for tasks to recognize facial expressions of basic emotions: a systematic review

**DOI:** 10.1590/1980-5764-dn-2022-0039

**Published:** 2022-12-05

**Authors:** Daiene de Morais Fabrício, Bianca Letícia Cavalmoretti Ferreira, Madson Alan Maximiano-Barreto, Monalisa Muniz, Marcos Hortes Nisihara Chagas

**Affiliations:** 1 Universidade Federal de São Carlos, Departamento de Psicologia, São Carlos SP, Brazil.; 2 Universidade Federal de São Carlos, Grupo de Pesquisa em Saúde Mental, Cognição e Envelhecimento, São Carlos SP, Brazil.; 3 Universidade de São Paulo, Departamento de Neurociências e do Comportamento, Ribeirão Preto SP, Brazil.; 4 Universidade Federal de São Carlos, Grupo de Pesquisa de Inteligência Emocional, São Carlos SP, Brazil.; 5 Instituto Bairral de Psiquiatria, Itapira SP, Brazil.

**Keywords:** Facial Expression, Validation Study, Emotions, Facial Recognition, Psychometrics, Expressão Facial, Estudo de Validação, Emoções, Reconhecimento Facial, Psicometria

## Abstract

Recognizing the other's emotions is an important skill for the social context that can be modulated by variables such as gender, age, and race. A number of studies seek to elaborate specific face databases to assess the recognition of basic emotions in different contexts.

**Objectives:**

This systematic review sought to gather these studies, describing and comparing the methodologies used in their elaboration.

**Methods:**

The databases used to select the articles were the following: *PubMed, Web of Science, PsycInfo,* and *Scopus*. The following word crossing was used: *“Facial expression database* OR *Stimulus set* AND *development* OR *Validation.”*

**Results:**

A total of 36 articles showed that most of the studies used actors to express the emotions that were elicited from specific situations to generate the most spontaneous emotion possible. The databases were mainly composed of colorful and static stimuli. In addition, most of the studies sought to establish and describe patterns to record the stimuli, such as color of the garments used and background. The psychometric properties of the databases are also described.

**Conclusions:**

The data presented in this review point to the methodological heterogeneity among the studies. Nevertheless, we describe their patterns, contributing to the planning of new research studies that seek to create databases for new contexts.

## RESUMO.

Reconhecer as emoções do outro é uma habilidade importante para o contexto social, que pode ser modulada por variáveis como sexo, idade e raça. Vários estudos buscam elaborar bancos de faces específicos para avaliar o reconhecimento de emoções básicas em diferentes contextos.

**Objetivos:**

Esta revisão sistemática buscou reunir esses estudos, descrevendo e comparando as metodologias utilizadas em sua elaboração.

**Métodos:**

As bases de dados utilizadas para a seleção dos artigos foram: PubMed, Web of Science, PsycInfo e Scopus. Foi utilizado o seguinte cruzamento de palavras: *“facial expression database* OR *stimulus set* AND *development* OR *validation”*.

**Resultados:**

O total de 36 artigos mostrou que a maioria dos estudos utilizou atores para expressar as emoções, que foram suscitadas de situações específicas para serem o mais espontâneas possível. Os bancos de faces foram compostos principalmente de estímulos coloridos e estáticos. Além disso, a maioria dos estudos buscou estabelecer e descrever padrões para registrar os estímulos, como a cor das roupas utilizadas e o fundo. As propriedades psicométricas dos bancos de faces também são descritas.

**Conclusões:**

Os dados apresentados nesta revisão apontam para a heterogeneidade metodológica entre os estudos. Apesar disso, descrevemos seus padrões, contribuindo para o planejamento de novas pesquisas que buscam criar bancos de faces específicos para novos contextos.

## INTRODUCTION

Emotions play an important role in society life, as they enable interaction among people. According to the evolutionary theories, all emotions derive from a set of basic emotions common to both humans and animals and which are genetically determined^1,2^. One of the ways for us to recognize the other's emotion is through facial expressions, since the face is one of the most expressive visual stimuli in society life^3^. The ability to recognize emotions through the face can already be perceived in newborns, a fact that justifies the innate nature of this skill^4^.

From a study using a systematized task, Ekman and Friesen^5^ postulated six basic emotions, which are related to evolutionary adaptations and can be universally recognized, namely, happiness, sadness, fear, disgust, surprise, and anger. In addition, they identified that the cultural aspects did not modulate the way in which these emotions were expressed^5^. Thus, the evidence indicated that all human beings had the same movements of the facial muscles under certain circumstances^6,7^, turning the ability to express emotions into a behavioral phenotype.

However, a number of studies began to notice that, within this phenotype common to human beings, some variables could modulate the way to recognize these facial expressions, such as cultural context^8^, age^9^, gender^10^, and race^11^. Taking these variables into account, several studies started to construct and validate specific face databases to assess the ability to recognize emotions through facial expressions^12–16^ since, when selecting a set of facial expression stimuli, it is necessary to consider characteristics of the model that are expressing the emotions, as well as who will recognize them.

Therefore, the existing facial expression databases present great diversity with regard to the physical characteristics of those who express the emotions, the way in which emotions are induced during the construction of the image database, and how they are presented in the validation stage^12–14^. Despite the methodological differences across the studies, they follow important standards for the construction and validation of the series of stimuli. Comparing the methodology used by the studies in the creation of these databases, regardless of the characteristics of who expresses the stimuli, can contribute to the planning of new research studies that seek to create face databases for new contexts. Thus, the objective of this systematic review was to gather studies that constructed face databases to assess the recognition of facial expressions of basic emotions, describing and comparing the methodologies used in the stimuli construction phase.

## METHODS

### Search strategies and eligibility criteria

The search strategy for this systematic review was created and implemented prior to study selection, in accordance with the checklist presented in the Preferred Reporting Items for Systematic Reviews and Meta-Analyses (PRISMA)^17^. The databases used to select the articles were the following: *PubMed, Web of Science, PsycInfo,* and *Scopus*. The following word crossing was used: *“Facial expression database* OR *Stimulus set* AND *development* OR *Validation.”* The searches were conducted from June to December 8, 2021.

The lists of references of the selected articles were also researched for additional sources. The inclusion criteria were surveys that constructed face databases to assess the recognition of basic emotions, published in original articles or disclosed on official websites, without language or time restrictions. Letters to the editor, books and book chapters, reviews, comments, notes, errata, theses, dissertations, and bibliographic/systematic reviews were excluded. In addition, it is worth noting that only the construction stage of the databases was included in this review.

Therefore, additional studies conducted after construction, such as normative data, were not contemplated in the analysis.

### Study selection

All the articles found in the databases were saved in the *Rayyan* electronic reference manager. After removing duplicate articles and according to the inclusion criteria of this study, all articles were evaluated by two independent researchers (DF and BF) through their titles and abstracts. In this stage, the researchers classified the articles as “yes,” “no,” or “perhaps.” Subsequently, the researchers reached consensus as to whether the articles recorded as “perhaps” should be included in the review.

After the inclusion of these studies, three researchers (DM, BF, and MB) read the articles in full and extracted information such as year of publication and study locus, name of the database built, characteristics of the participants who expressed the emotions (number of participants, place of recruitment, gender, age and race), basic emotions expressed, and final total of stimuli included in the database and their specific characteristics (Table 1)^12–16,25–63^. Subsequently, the methodological characteristics of the databases were collected, such as the method used to elicit the emotions, patterns in the capture of stimuli, criteria used in the validation stage, sample characteristics in the validation stage, and psychometric qualities assessed (Table 2)^12–16,25–63^.

**Table 1:** General characteristics of face databases.

Authors and year of publication	Study location	Name of the built database	Theoretical reference	Characteristics of participants who expressed emotions	Basic emotions expressed	Total of stimuli	Specific characteristics of stimuli
Benda and Scherf. (2020)^25^	The United States	Complex Emotion Expression Database (CEED)	Recognition of complex emotions in young people (Empirical)	8 professional actors – Age: 20.9 years; SD=3.1 – Sex: M=4; F=4 – Race: Caucasians (n=4) and Black (n=4)	1) Happiness 2) Sadness 3) Fear 4) Disgust 5) Anger 6) Surprise	243 images	– Black and white – Static
Chung et al. (2019)^26^	South Korea	Yonsei Face Database (YFace DB)	Basic emotions (Ekman and Friesen, 1975)^56^	74 local community and university volunteers – Age: 19-40 years – Sex: M=37; F=37 – Race: Koreans	1) Happiness 2) Sadness 3) Fear 4) Disgust 5) Anger 6) Surprise 7) Neutral	1,480 stimuli	– Colorful – Static and dynamic *Open and closed mouth *Varied intensities
Conley et al. (2018)^16^	The United States	The racially diverse affective expression (RADIATE)	Racial heterogeneity in emotion recognition (Empirical)	109 community adults – Age: 18-30 years – Sex: M=53; F=56 – Race: Asian (n=22), Black/African-Americans (n=38), Caucasians (n=28), Hispanic (n=20) and others (n=1)	1) Happiness 2) Sadness 3) Fear 4) Disgust 5) Anger 6) Surprise 7) Neutral	1,721 images	– Colorful and black and white – Static *Open and closed mouth
Dalrymple et al. (2013)^27^	The United States	The Dartmouth Database of Children's Faces	Recognition of emotions in children (Empirical)	80 community children – Age: 9.84 years; SD=2.33 – Sex: M=40; F=40 – Race: Caucasians	1) Happiness 2) Sadness 3) Fear 4) Disgust 5) Anger 6) Surprise 7) Neutral	964 images	– Colorful – Static *Happiness with closed mouth and happiness showing teeth
Donadon et al. (2019)^28^	Brazil	Baby Faces	Ekman's Neurocultural Theory (1972)^57^	20 babies – Age: 9 months; SD=1.5 – Sex: M=10; F=10 – Race: Caucasians (n=66%), Black (n=17%), and Japanese (n=17%)	1) Happiness 2) Sadness 3) Fear 4) Anger 5) Surprise 6) Neutral	57 images	– Colorful – Static
Ebner et al. (2010)^13^	Germany	Faces--a life-span Database of Facial Expressions	Age differences in emotion recognition (Ruffman et al., 2008)^58^	179 actors and extras recruited from a modeling agency – 61 young (24.3 years; SD=3.5) – 60 middle-age (49.0 years; SD=3.9) – 58 elderly (73.2 years; SD=2.8) – Sex: M=86; F=85 – Race: Caucasians (n=179)	1) Happiness 2) Sadness 3) Fear 4) Disgust 5) Anger 6) Neutral	2,052 images	– Colorful – Static
Egger et al. (2011)^29^	The United States	NIMH Child Emotional Faces Picture Set (NIMH-ChEFS)	Recognition of emotions in children (Empirical)	59 child actors – Age: 13.6 years – Sex: M=20; F=39 – Race: ND	1) Happiness 2) Sadness 3) Fear 4) Anger 5) Neutral	482 images	– Colorful – Static *Two directions of gazing: direct and avoided
Ekman and Friesen. (1976)^30^	The United States	Pictures of Facial Affect (POFA)	Pan-cultural elements in facial expressions of emotions (Ekman et al., 1969)^5^	10 individuals – Age: ND – Sex: M=4; F=6 – Race: Caucasians and African-American	1) Happiness 2) Sadness 3) Fear 4) Disgust 5) Anger 6) Surprise 7) Neutral	110 images	– Black and white – Static
Fujimura and Umemura. (2018)^31^	Japan	A facial expression database based on the dimensional and categorical model of emotions	The influence of angles on emotion recognition (Borod et al., 1998)^59^	8 professional actors – Age: 34.25 years; SD=5.47 – Sex: M=4; F=4 – Race: Japanese	1) Happiness 2) Sadness 3) Fear 4) Disgust 5) Anger 6) Surprise 7) Neutral	920 stimuli	– Colorful – Static and dynamic *Open and closed mouth *Varied angles
Franz et al. (2021)^32^	Germany	Picture-Set of Young Children's Affective Facial Expressions (PSYCAFE)	Recognition of emotions in children (Empirical)	35 children – Age: 4-6 years – Sex: M=14; F=21 – Race: ND	1) Happiness 2) Sadness 3) Fear 4) Disgust 5) Anger 6) Surprise 7) Neutral	104 images	– Colorful – Static *Varied intensities
Garrido et al. (2017)^33^	Portugal	Stills and Videos of facial Expressions (SAVE database)	Recognition of emotions in dynamic stimuli (Empirical)	20 students – Age: 21.75 years; SD=1.97 – Sex: M=12; F=8 – Race: ND	1) Happiness 2) Neutral	120 stimuli	– Colorful – Static and dynamic
Giuliani et al. (2017)^15^	The United States	The DuckEES child and adolescent dynamic facial expressions stimulus set	Recognition of emotions in dynamic stimuli (Empirical)	37 children and teenage actors – Age: 13.24 years; SD=2.09 – Sex: M=15; F=22 – Race: Caucasians (n=89%)	1) Happiness 2) Sadness 3) Fear 4) Disgust 5) Neutral	120 videos	– Colorful – Dynamic
Happy et al. (2015)^34^	India	The Indian Spontaneous Expression Database for Emotion Recognition (ISED)	Basic emotions (Ekman and Friesen, 1975)^56^	50 individuals – Age: 18-22 years – Sex: M=29; F=21 – Race: Indians	1) Happiness 2) Sadness 3) Disgust 4) Surprise	428 videos	– Colorful – Dynamic *Varied intensities
Kaulard et al. (2012)^35^	Germany	The MPI Facial Expression Database	Language and emotions (Empirical)	19 native Germans without professional acting experience – Age: 20-30 years – Sex: M=9; F=10 – Race: Caucasians	1) Happiness 2) Sadness 3) Fear 4) Disgust 5) Anger	18800 videos	– Colorful – Dynamic *Varied angles
Keutmann et al. (2015)^36^	The United States	Visual and vocal emotional expressions of adult and child actors	Item Response Theory in face database construction (Empirical)	150 actors (Adults: n=139 and kids: n=11) – Age: 36.1 years; SD=15.6 – Sex: M=73; F=77 – Race: Caucasians (n=98), African-American (n=35), Hawaiian (n=1), mixed (n=1), and others (n=1)	1) Happiness 2) Sadness 3) Fear 4) Anger 5) Neutral	152 stimuli	– Colorful – Static and dynamic *Varied intensities
Kim et al. (2017)^37^	South Korea	Korea University Facial Expression Collection – Second Edition (KUFEC-II)	The role of culture in recognizing emotions (Empirical)	57 actors – Age: ND – Sex: M=32; F=36 – Race: Koreans	1) Happiness 2) Sadness 3) Fear 4) Disgust 5) Anger 6) Surprise 7) Neutral	399 images	– Colorful – Static
Langner et al. (2010)^38^	Netherlands	Radboud Faces Database	The influence of angles and direction of gaze on emotion recognition (Empirical)	49 young and children Young: 39 Children: 10 – Age: ND – Sex: M=24; M=25 – Race: Caucasians (n=49)	1) Happiness 2) Sadness 3) Fear 4) Disgust 5) Anger 6) Surprise 7) Neutral	5,880 images	– Colorful – Static *Three directions of gaze: front, right, and left *Varied face angles
LoBue and Thrasher. (2015)^14^	The United States	The Child Affective Facial Expression (CAFE)	Recognition of emotions in children's faces of different races (Empirical)	154 children – Age: 5.3 years – Sex: M=64; F=90 – Race: African-Americans (n=27), Caucasians (n=77), Asians (n=16), Latinos (n=23), and South Asia (n=11)	1) Happiness 2) Sadness 3) Fear 4) Disgust 5) Anger 6) Surprise 7) Neutral	1,192 images	– Colorful – Static *Open and closed mouth
Lundqvist et al. (1998)^39^	Sweden	Karolinska Directed Emotional Faces (KDEF) Database	-	70 actors – Age: 25 years (20-30 years) – Sex: M=35; F=35 – Race: ND	1) Happiness 2) Sadness 3) Fear 4) Disgust 5) Anger 6) Surprise 7) Neutral	490 images	– Colorful – Static *Varied face angles
Ma et al. (2020)^40^	China	Han, Hui, and Tibetan Chinese facial expression database	The role of culture in recognizing emotions (Empirical)	630 volunteers – Age: Han (22 years; SD=2.7); Hui (22.8 years; SD=2.4); and Tibet (21.4 years; SD=2.5) – Sex: M=315; F=315 – Race: Chinese from different regions	1) Happiness 2) Sadness 3) Fear 4) Disgust 5) Anger 6) Surprise 7) Neutral	930 images	– Colorful – Static
Ma et al. (2015)^41^	The United States	Chicago Face Database (CFD)	Limitations of existing face databases (Empirical)	158 individuals from the University of Chicago Laboratory and amateur actors – Age: 13.6 years – Sex: M=73; F=85 – Race: Black (n=85) and Caucasians (n=73)	1) Happiness 2) Fear 3) Neutral	158 images	– Colorful – Static *Two directions of gaze: direct and averted
Maack et al. (2017)^42^	Norway	The Tromso Infant Faces Database (TIF)	Influence of child stimuli on the adult attention system (Brosch et al., 2007; Parsons et al., 2011; Borgi et al., 2014)^60-62^	18 babies – Age: 4-12 months – Sex: M=8; F=10 – Race: Caucasians	1) Happiness 2) Sadness 3) Fear 4) Disgust 5) Anger 6) Surprise 7) Neutral	119 images	– Colorful – Static
Meuwissen et al. (2017)^43^	The United States	Developmental Emotional Faces Stimulus Set (DEFSS)	Limitations of existing face databases (Empirical)	116 volunteers 42 children 44 teenagers 30 adults – Age: ND – Sex: M=43; F=73 – Race: White (n=102), non-White (n=15)	1) Happiness 2) Sadness 3) Fear 4) Anger 5) Neutral	404 images	– Colorful – Static
Minear and Park. (2004)^44^	The United States	A lifespan database of adult facial stimuli	Influence of age on emotion recognition (Empirical)	576 community volunteers – Age: 18-93 years – Sex: M=219; F=357 – Race: Caucasians (n=435), African-American (n=89), and others (n=52)	1) Happiness 2) Neutral	1,142 images	– Colorful – Static
Negrão et al. (2021)^45^	Brazil	The Child Emotion Facial Expression Set	Recognition of emotions in children (Empirical)	132 children – Age: 4-6 years – Sex: M=42%; F=58% – Race: Caucasian (n=71%), African (n=24%), Asian (5%)	1) Happiness 2) Sadness 3) Fear 4) Disgust 5) Anger 6) Surprise 7) Neutral	971 stimuli	– Colorful – Static and dynamic
Novello et al. (2018)^46^	Brazil	Youth Emotion Picture Set	Recognition of Facial Emotions in Teens (Empirical)	31 randomly selected volunteers – Age: 17.4 years; SD=2.7 – Sex: M=14; F=17 – Race: Caucasians (n=27), Blacks (n=1), and mixed (n=3)	1) Happiness 2) Sadness 3) Fear 4) Disgust 5) Anger 6) Surprise 7) Neutral	42 images	– Black and white – Static
O'Reilly et al. (2016)^47^	The United Kingdom	The EU-Emotion Stimulus Set	Limitations of existing face databases (Empirical)	19 actors – Age: 10-70 years – Sex: M=9; F=10 – Race: Caucasians (n=13), Afro-Caribbean/British-Asian (n=2), Blacks (n=2), mixed white/Asian (n=1), Mediterranean/Asian-British (n=1)	1) Happiness 2) Sadness 3) Fear 4) Disgust 5) Anger 6) Surprise 7) Neutral	249 videos	– Colorful – Dynamic
Olszanowski et al. (2015)^48^	Poland	Warsaw set of emotional facial expression. pictures (WSEFEP)	Limitations of existing face databases (Empirical)	30 professional actors – Age: 20-30 years – Sex: M=14; F=16 – Race: Polish	1) Happiness 2) Sadness 3) Fear 4) Anger 5) Surprise 6) Neutral	210 images	– Colorful – Static
Passareli et al. (2018)^49^	Italy	Facial Expression Recognition Test (FERT)	Basic emotions (Ekman e Friesen, 1975)^56^ and Item Response Theory (Reise and Revicki, 2014)^63^	6 professional actors – Age: ND – Sex: M=3; F=3 – Race: ND	1) Happiness 2) Sadness 3) Fear 4) Disgust 5) Anger 6) Surprise 7) Neutral	42 images	– Colorful – Static
Romani-Sponchiado et al. (2015)^50^	Brazil	Child Emotions Picture Set	Recognition of facial emotions in children (Empirical)	18 children – Age: 6-7 years (6.93 years; SD=0.3); 8-9 years (9.12 years; SD=0.57), and 10-11 years (10.72 years; SD=0.61) – Sex: M=9; F=9 – Race: Caucasians (n=14), African-American (n=3), and Indigenous (n=1)	1) Happiness 2) Sadness 3) Fear 4) Disgust 5) Anger 6) Surprise 7) Neutral	225 images	– Black and white – Static *Varied intensities
Samuelsson et al. (2012)^51^	Sweden	Umeå University Database of Facial Expressions	Limitations of existing face databases (Empirical)	60 community individuals – Age: 17-67 years (30.19 years; SD=10.66) – Sex: M=30; F=30 – Race: Swedes, Central Europe, Arabs, and Asians	1) Happiness 2) Sadness 3) Fear 4) Disgust 5) Anger 6) Surprise 7) Neutral	424 images	– Colorful – Static
Sharma and Bhushan. (2019)^52^	India	Indian Affective Picture	Basic emotions (Ekman and Friesen, 1975)^56^ and limitations of existing face databases (Empirical)	4 professional actors – Age: 25.25 years; SD=3.77 – Sex: M=2; F=2 – Race: Indians	1) Happiness 2) Sadness 3) Fear 4) Disgust 5) Anger 6) Surprise 7) Neutral	140 images	– Colorful – Static *Varied face angles
Tottenham et al. (2009)^12^	The United States	The NimStim set of facial expressions	Basic emotions (Ekman and Friesen, 1975)^56^ and limitations of existing face databases (Empirical)	43 professional actors – Age: 21-30 years – Sex: M=25; F=18 – Race: Africans, Europeans, and Latin Americans	1) Happiness 2) Sadness 3) Fear 4) Disgust 5) Anger 6) Surprise 7) Neutral	672 images	– Colorful – Static *Open and closed mouth
Tracy et al. (2009)^53^	Canada	Universidade da Califórnia, Davis, Set of Emotion Expressions (UCDS)	Basic emotions (Ekman and Friesen, 1975)^56^ and limitations of existing face databases (Empirical)	28 community individuals – Age: 27.0 years – Sex: M=14; F=14 – Race: White and African	1) Happiness 2) Sadness 3) Fear 4) Disgust 5) Anger 6) Surprise	73 images	– Colorful – Static
Vaiman et al. (2017)^54^	Argentina	Expresiones de Emociones Faciales (FACS)	The role of culture in recognizing emotions (Empirical)	14 Argentines from the community – Age: 25.53 years; SD=8.72 – Sex: M=8; F=6 – Race: ND	1) Happiness 2) Sadness 3) Fear 4) Disgust 5) Anger 6) Surprise 7) Neutral	60 images	– Colorful – Static
Yang et al. (2020)^55^	China	Tsinghua facial expression database	The role of culture in recognizing emotions (Empirical)	63 young and 47 elderly Chinese natives with an interest in acting Young – Age: 23.82 years; SD=4.18 – Sex: M=32; F=31 – Race: Chinese Elderly – Age: 64.40 years; SD=3.51 – Sex: M=21; F=26 – Race: Chinese	1) Happiness 2) Sadness 3) Fear 4) Disgust 5) Anger 6) Surprise 7) Neutral	880 images	– Colorful – Static

ND: not declared; M: male; F: female; SD: standard deviation.

* Additional features of the face database.

**Table 2:** Methodological characteristics used in the studies to create the databases.

Authors and year of publication	Name of the database elaborated	Method used to elicit the emotions	Patterns in stimulus capture	Criteria used in the validation stage for inclusion of stimuli in the final database	Sample characteristics in the stage for the validation of the stimuli	Psychometric properties assessed
Benda and Scherf. (2020)^25^	Complex Emotion Expression Database (CEED)	1) Presentation of an equivalent photograph expressing the emotion 2) Emotions elicited from specific situations	– Background: White – Clothes: ND – Distractors removed: ND	Accuracy ≥50%	796 volunteers recruited through MTurk – Age: 34. years; SD=11.6 – Gender: M=403; F=388 – Race: ND	– Analysis of the items: Accuracy* – Validity evidence: Content-based: Accuracy and error in each item
Chung et al. (2019)^26^	Yonsei Face Database (YFace DB)	1) Presentation of an equivalent photograph expressing the emotion 2) Instruction on muscle movement of the emotions based on the FACS 3) Emotions elicited from specific situations	– Background: White – Clothes: Black T-shirt – Distractors removed: Beards, glasses, makeup, and bangs	Accuracy, intensity, and naturalness	212 students from the Seoul University – Age: 18-28 years – Gender: M=97; F=115 – Race: ND	– Analysis of the items: Accuracy† – Precision: Accuracy – Validity evidence: Content-based: Accuracy Based on the relationship with other variables: ANOVA for difference in precision between genders of the stimuli and evaluators, t-test for difference in mean accuracy between genders and emotions, and post-hoc Bonferroni analysis for items with significant differences‡
Conley et al. (2018)^16^	The racially diverse affective expression (RADIATE)	Presentation of an equivalent photograph expressing the emotion	– Background: White – Clothes: White sheet – Distractors removed: Glasses, headband, hats	Accuracy and Cohen's kappa	662 participants recruited through MTurk – Age: 18-35 years (27.6 years; SD=3.8) – Gender: M=402; F=260 – Race: Asian (n=48), Black/African-American (n=70), Caucasian (n=470), Hispanic (n=63), and others (n=11)	– Precision: Reliability (test-retest)† – Validity evidence: Content-based: Accuracy†; Cohen's kappa† and variability in precision by race of the model
Dalrymple et al. (2013)^27^	The Dartmouth Database of Children's Faces	Emotions elicited from specific situations	– Background: Black – Clothes: Black dresses and black hats – Distractors removed: Glasses and jewelry	Images recognized with ≥70% accuracy	163 students and members of the Dartmouth College academic community – Age: 19.6 years; SD=4.15 – Gender: M=67; F=96 – Race: ND	– Precision: Accuracy† and Cohen's kappa among the evaluators† – Validity evidence: Content-based: Accuracy and Cohen's kappa among the evaluators Based on the relationship with other variables: ANOVA for difference in precision between gender of the stimuli and evaluators‡
Donadon et al. (2019)^28^	Baby Faces	The parents were instructed and trained to provoke the intended emotions	ND	Rasch model to minimize floor and ceiling effects with values from 0.50 to 1.50 Rate of correct answers according to Kringelbach et al. 2008^64^	Validation 119 volunteers from the community – Age: 36 years; SD=12.8 – Gender: M=36.1%; F=63.9% – Race: Caucasian (n=69.7%), Black (n=26.1%), and Japanese (n=4.2%) Retest 31 volunteers from the community – Age: 38.06 years; SD=11.57 – Gender: M=35.5%; F=64.5% – Race: Caucasian (n=74%), Black (n=19.5%), and Japanese (n=6.5%)	– Analysis of the items: Adjustment and difficulty of the items by the Rasch model – Precision: Reliability (test-retest)† – Validity evidence: Content-based: Accuracy§ Based on the relationship with other variables: ANCOVA to assess the differences between groups considering the sociodemographic variables (gender, race, schooling level of the adults, and gender and race of the faces in the stimulus)‡
Ebner et al. (2010)^13^	Faces--a life-span Database of Facial Expressions	1) Emotion induction through photographs and videos 2) Emotions elicited from specific situations	– Background: Gray – Clothes: Gray T-shirt – Distractors removed: Jewelry, glasses, and makeup	Agreement among evaluators for (1) purity of the facial expression and (2) high intensity facial expression	154 students – Age: 20-81 years – Gender: M=78; F=76 – Race: Caucasian	– Precision: Accuracy† and consensus among the evaluators† – Validity evidence: Content-based: Accuracy and consensus among the evaluators Based on the relationship with other variables: ANOVA for face age × evaluator's age × emotion expressed‡
Egger et al. (2011)^29^	NIMH Child Emotional Faces Picture Set (NIMH-ChEFS)		– Background: Gray – Clothes: ND – Distractors removed: ND	The cutoff point for the image to be included was that ≥15 evaluators identified the intended emotion	20 professors and employees of the Duke University Medical Center – Age: 38.3 years – Gender: M=7; F=13 – Race: ND	– Analysis of the items: Accuracy† – Difficulty of the items: Intensity and representativeness scores – Precision: Agreement among the evaluators^//^ – Validity evidence: Content-based: Accuracy and agreement among the evaluators
Ekman and Friesen. (1976)^30^	Pictures of Facial Affect (POFA)	Instruction on muscle movement of the emotions based on FACS	ND	ND	ND	ND
Fujimura and Umemura (2018)^31^	A facial expression database based on the dimensional and categorical model of emotions	1) Emotions elicited from specific situations 2) Instruction on muscle movement of the emotions based on FACS	– Background: White – Clothes: White T-shirt – Distractors removed: Glasses and strong makeup	Agreement among the evaluators Mean of 69% agreement among the evaluators (SD=21%)	39 university students – Age: 21.33 years; SD=2.39 – Gender: M=19; F=20 – Race: Japanese natives	– Precision: Accuracy† – Validity evidence: Content-based: Accuracy and confusion matrix of agreement rates for images of dynamic and static expressions of each model
Franz et al. (2021)^32^	Picture-Set of Young Children's Affective Facial Expressions (PSYCAFE)	1) Guidance of emotions in theater workshops 2) Directed Facial Action Task used to guide the movement of anatomical landmarks	– Background: White – Clothes: ND (just face) – Distractors removed: ND (just face)	Step 1 Confirmatory hierarchical cluster analysis by Ward Step 2 Intensity, authenticity, and likeability. Accuracy (77-100%) and AFFDEX Software	Step 1 197 volunteers from the community – Age: 32.9 years; SD=16.1 – Gender: M=33%; F=67% – Race: ND Step 2 44 volunteers from the community – Age: 25.7 years; SD=5.9) – Gender: M=48%; F=52% – Race: ND	– Precision: Accuracy† – Validity evidence: Based on the relationship with other variables: Stimulus age × expressed emotion × accuracy
Garrido et al. (2017)^33^	Stills and Videos of facial Expressions (SAVE database)	Emotions elicited from specific situations	– Background: Gray – Clothes: White T-shirt – Distractors removed: Jewelry, glasses, and makeup	Stimuli with an assessment of 2.5 SD above or below the mean	120 university students – Age: 20.62 years; SD=3.39 – Gender: M=22.5%; F=77.5% – Race: Caucasian	– Precision: Accuracy† – Validity evidence: Content-based: Accuracy and interest dimensions (valence, excitement, clarity, intensity, appeal, similarity, and familiarity) Based on the relationship with other variables: Accuracy × gender of the model and the participant¶
Giuliani et al. (2017)^15^	The DuckEES child and adolescent dynamic facial expressions stimulus set	Emotions elicited from specific situations	– Background: White – Clothes: ND – Distractors removed: ND	Images recognized with ≥70% accuracy	36 volunteers from the Oregon University – Age: 19.5 years; SD=1.95 – Gender: M=14; F=22 – Race: ND	– Precision: Accuracy† – Validity evidence: Content-based: Accuracy and Fleiss’ kappa†
Happy et al. (2015)^34^	The Indian Spontaneous Expression Database for Emotion Recognition (ISED)	Emotion induction through videos	– Background: ND – Clothes: ND – Distractors removed: ND	Agreement among the evaluators (Fleiss’ Kappa)	Four trained evaluators – Age: ND – Gender: M=2; F=2 – Race: ND	– Precision: Accuracy† – Validity evidence: Content-based: Accuracy and Fleiss’ kappa†
Kaulard et al. (2012)^35^	The MPI Facial Expression Database	Emotions elicited from specific situations	– Background: Black – Clothes: Black cape and hats – Distractors removed: Makeup and beards	Consistency among the evaluators (Fleiss’ Kappa)	20 German natives – Age: 19-33 years – Gender: M=10; F=10 – Race: ND	– Precision: Accuracy† – Validity evidence: Content-based: Accuracy and Fleiss’ kappa†
Keutmann et al. (2015)^36^	Visual and vocal emotional expressions of adult and child actors	Emotions elicited from specific situations	– Background: Green – Clothes: ND – Distractors removed: ND	Accuracy	510 students, 226 from Drexel University and 284 from the University of Central Florida – Age: ND – Gender: ND – Race: ND	– Analysis of the items: Difficulty analysis and item discrimination by means of the classical test theory – Precision: Accuracy† – Validity evidence: Content-based: Accuracy
Kim et al. (2017)^37^	Korea University Facial Expression Collection – Second Edition (KUFEC-II)	Instruction on muscle movement of the emotions based on FACS	– Background: Gray – Clothes: Pattern – Distractors removed: Makeup, accessories, and dyed hair	Internal consistency Accuracy	75 evaluators – Age: 19-69 years (26.17 years, SD=5.69) – Gender: M=39; F=36 – Race: ND	– Precision: Accuracy† – Validity evidence: Content-based: Accuracy; agreement among the evaluators† and scores for purity, valence, and intensity Based on the relationship with other variables: ANOVA to test the effects of gender on recognition‡ and correlations between the participant's emotional state and task performance¶
Langner et al. (2010)^38^	Radboud Faces Database	Instruction on muscle movement of the emotions based on FACS	– Background: White – Clothes: Black T-shirt – Distractors removed: Glasses, earrings and makeup	Accuracy	276 students from Radboud University – Age: 21.2 years; SD=4.0 – Gender: M=38; F=238 – Race: ND	– Precision: Accuracy† – Validity evidence: Content-based: Accuracy and dimensions of interest (type of expression, intensity, clarity, genuineness, and valence) Based on the relationship with other variables: ANOVA comparing each of the precision variables with age, gender, expression, and gaze direction‡
LoBue and Thrasher. (2015)^14^	The Child Affective Facial Expression (CAFE)	Instruction on muscle movement of the emotions based on FACS was carried out during improvised games	– Background: White – Clothes: White sheet – Distractors removed: ND	Images recognized with ≥60% accuracy	– 100 undergraduate students from Rutgers University – Age: ND – Gender: M=50; F=50 – Race: African-American (n=17%), Asian (n=27%), White (n=30%), Latin (n=17%), and others (n=9%)	– Analysis of the items: Difficulty of the items: Rasch model – Precision: Test-retest reliability† and accuracy# – Validity evidence: Content-based: Accuracy
Lundqvist et al. (1998)^39^	Karolinska Directed Emotional Faces (KDEF) Database	The participants were free to express the emotion as they wished	Background: Neutral Clothes: Gray T-shirt Distractors removed: Beard, mustache, earrings, glasses, and makeup	ND	ND	ND
Ma et al. (2020)^40^	Han, Hui, and Tibetan Chinese facial expression database	1) Emotion induction through photographs and videos 2) Instruction on muscle movement of the emotions based on FACS	– Background: Black – Clothes: ND – Distractors removed: Jewelry	Images recognized with ≥60% accuracy	– 240 volunteers (80 from each study region) – Age: 23 years; SD=1.7 – Gender: M=120; F=120 – Race: Chinese	– Precision: Accuracy** and method of halves – Validity evidence: Content-based: Accuracy Based on internal consistency: Cronbach's alpha†
Ma et al. (2015)^41^	Chicago Face Database (CFD)	1) Emotions expressed from verbal instructions 2) Presentation of an equivalent photograph expressing the emotion	– Background: White – Clothes: Gray T-shirt – Distractors removed: ND	Two independent judges assessed how believable the expression was on a Likert scale from 1 to 9 (1=not at all believable; 9=very believable)	1,087 evaluators (convenience sample) – Age: 26.7 years; SD=10.5 – Gender: M=308; F=552 – Race: White (n=516), Asian (n=117), Black (n=74), bi–or multi-race (n=72), Latin (n=57), others (n=18), and did not report (n=233)	– Precision: Accuracy – Validity evidence: Based on the internal structure: exploratory factor analysis (Varimax rotation) Content-based: Accuracy; agreement among the evaluators† and effects of race and gender of the stimuli (criteria for item construction)
Maack et al. (2017)^42^	The Tromso Infant Faces Database (TIF)	The parents were instructed to elicit the intended emotions with games and specific stimuli	– Background: White – Clothes: White overalls and hat – Distractors removed: ND	The photographs with best agreement among the evaluators were selected Mean classification of clarity and intensity below 2.5 Validation: (a) expression portrayed, (b) clarity of expression, (c) intensity of the expression, and (d) valence of the expression	720 participants – Age: 18-70 years (32.8 years; SD=10.4) – Gender: M=21%; F=79% – Race: ND	– Precision: Accuracy†† – Validity evidence: Content-based: dimensions of interest (type of expression, clarity, intensity, and valence) Based on the relationship with other variables: ANOVA to compare performance × child-rearing stage × gender × mood¶
Meuwissen et al. (2017)^43^	Developmental Emotional Faces Stimulus Set (DEFSS)	1) Emotions elicited from specific situations 2) Presentation of an equivalent photograph expressing the emotion	– Background: Gray – Clothes: ND – Distractors removed: Jewelry	The images recognized by less of 55% of the evaluators were excluded	228 university students between undergraduate and graduate levels and children preappointed by the family via the Internet – Age: 8-30 years – Gender: M=150; F=254 Race: White (n=81%), non-White (n=17%)	– Precision: Accuracy‡‡ – Validity evidence: Content-based: correct answers by age group, intensity, and emotion
Minear and Park. (2004)^44^	A life span database of adult facial stimuli	Emotions expressed from verbal instructions	– Background: Gray – Clothes: ND – Distractors removed: ND	ND	ND	ND
Negrão et al. (2021)^45^	The Child Emotion Facial Expression Set	1) Presentation of an equivalent photograph expressing the emotion 2) Emotions elicited from specific situations	– Background: White – Clothes: White – Distractors removed: ND	Step 1: 100% agreement between two evaluators Step 2: 100% agreement between other two evaluators (two of each step)	Four judges – Age: ND – Gender: ND – Race: ND	– Precision: Accuracy† and Cohen's kappa† – Validity evidence: Based on the relationship with other variables: accuracy × gender × age¶; emotion × race‡
Novello et al. (2018)^46^	Youth Emotion Picture Set	1) Emotions elicited from specific situations 2) Presentation of an equivalent photograph expressing the emotion 3) Presentation of videos and a game to specifically elicit the emotion of anger	– Background: ND – Clothes: Black cape – Distractors removed: Jewelry	Images recognized with ≥75% accuracy	Adults: 101 volunteers recruited through the snowball method – Age: 18-77 years – Gender: M=31.7%; F=68.3% – Race: ND Adolescents: 54 volunteers from state schools – Age: 12-17 years – Gender: M=40.7%; F=59.3% – Race: ND	– Precision: Accuracy† and Cohen's kappa† – Validity evidence: Based on the relationship with other variables: comparison of performance by age¶
O'Reilly et al. (2016)^47^	The EU-Emotion Stimulus Set	Emotions elicited from specific situations	– Background: White – Clothes: ND – Distractors removed: ND	Accuracy	1,231 volunteers – Age: 44 years; SD=16.7 – Gender: M=428; F=803 – Race: ND	– Precision: Accuracy_ and Cohen's kappa† – Validity evidence: Content-based: performance comparison by expression type, valence, and excitation
Olszanowski et al. (2015)^48^	Warsaw Set of Emotional Facial Expression Pictures (WSEFEP)	Instruction on muscle movement of the emotions based on FACS	– Background: White – Clothes: Black T-shirt – Distractors removed: Beards, mustaches, earrings, and glasses	Agreement in recognition	1,362 participants – Age: 26.6 years; SD=11.6 – Gender: M=261; F=1,101 – Race: ND	– Precision: agreement among the evaluators – Validity evidence: Content-based: purity analysis and intensity coefficient
Passareli et al. (2018)^49^	Facial Expression Recognition Test (FERT)	Presentation of an equivalent photograph expressing the emotion	– Background: Black – Clothes: Black T-shirt – Distractors removed: ND	Unidimensional model	794 volunteers from the community – Age: 36.13 years; SD=13.79 – Gender: M=36.2%; F=63.8% – Race: ND	– Validity evidence: Based on the internal structure: factor analysis through the two-parameter Bayesian model – Based on the relationship with other variables; performance comparison between gender and age‡ – Analysis of the items: Discrimination and difficulty through the Item Response Theory (IRT)
Romani-Sponchiado et al. (2015)^50^	Child Emotions Picture Set	Emotion induction through videos	– Background: ND – Clothes: ND – Distractors removed: ND	Images recognized with ≥60% accuracy	30 psychologists with experience in child development – Age: ND – Gender: ND – Race: ND	– Precision: Accuracy** and Fleiss’ Kappa† – Analysis of the items: Accuracy – Validity evidence: Content-based: Fleiss’ kappa; chi-square to compare the proportion of posed and spontaneous photographs
Samuelsson et al. (2012)^51^	Umeå University Database of Facial Expressions	Instruction on muscle movement of the emotions based on FACS	– Background: ND – Clothes: ND – Distractors removed: Makeup	Accuracy	526 participants – Age: 18-73 years (37.7 years; SD=13.0) – Gender: M=157; F=369 – Race: ND	– Precision: Accuracy† – Validity evidence: – Based on the relationship with other variables; performance comparison by gender and age¶
Sharma and Bhushan. (2019)^52^	Indian Affective Picture	1) Presentation of an equivalent photograph expressing the emotion 2) Emotions elicited from specific situations	– Background: ND – Clothes: ND – Distractors removed: Beards, glasses, and makeup	Accuracy Intensity (9-point scale)	350 undergraduate students – Age: 20.58 years; SD=1.13 – Gender: M=320; F=30 – Race: ND	– Analysis of the items: Accuracy† – Validity evidence: Based on the relationship with other variables: t-test to compare men's and women's performance¶
Tottenham et al. (2009)^12^	The NimStim set of facial expressions	Emotions expressed from verbal instructions	– Background: ND – Clothes: ND – Distractors removed: Makeup	Validity (accuracy and Cohen's kappa) and reliability	Group 1 47 university students – Age: 19.4 years (SD=1.2) – Gender: M=39; F=47 – Race: European-American (81%), African-American (6%), Asian-American (9%), and Hispanic-American (4%) Group 2 34 volunteers from the community – Age: 25.8 years (SD=4.1) – Gender: M=22; F=12 – Race: European-American (59%), African-American (18%), Asian-American (6%), Hispanic-American (6%), and other races (12%)	– Precision: Accuracy† and test-retest† – Validity evidence: Content-based: Accuracy and test-retest
Tracy et al. (2009)^53^	University of California, Davis, Set of Emotion Expressions (UCDS)	Instruction on muscle movement of the emotions based on FACS	– Background: Gray – Clothes: White T-shirt – Distractors removed: Jewelry	Accuracy (the most recognized emotion of each expression was included in the final database)	Study 1 175 undergraduate students – Age: ND – Gender: M=35%; F=65% – Race: ND Study 2 234 undergraduate students – Age: ND – Gender: M=21%; F=79% – Race: ND	– Analysis of the items: Accuracy§§ – Validity evidence: Content-based: Accuracy and performance based on race and gender of stimulus
Vaiman et al. (2017)^54^	FACS	Emotions elicited from specific situations	– Background: Blue – Clothes: White T-shirt – Distractors removed: Hair back (hair up)	Images recognized with ≥70% accuracy	466 students from the Psychology School of the National University of Córdoba. – Age: 20.29 years; SD=4.33 – Gender: M=23%; F=79% – Race: ND	– Precision: Accuracy† – Analysis of the items: Discrimination – Validity evidence: Based on the convergent relationship: Descriptive comparison of database performance vs. POFA database performance†
Yang et al. (2020)^55^	Tsinghua facial expression database	1) Emotions elicited from specific situations 2) Instruction on muscle movement of the emotions based on FACS	– Background: White – Clothes: ND – Distractors removed: Tattoos, piercings, jewelry, glasses, and makeup.	Images recognized with ≥70% accuracy	34 young individuals and 31 older adults, Chinese Young individuals – Age: 19-35 years (23.50 years; SD=4.41) – Gender: M=19; F=15 – Race: Chinese Older adults – Age: 58-72 years (65.06 years; SD=3.50) – Gender: M=13; F=18 – Race: Chinese	– Precision: Accuracy† and kappa agreement among the evaluators† – Validity evidence: Content-based: Accuracy and kappa agreement among the evaluators

ND: not declared; M: male; F: female; MTurk: Amazon Mechanical Turk; FACS: Facial Action Coding System (Ekman and Friesen, 1978)^65^; ANCOVA: analysis of covariance; ANOVA: repeatedmeasure analysis of variance.

* Only images with ≥50% accuracy were included in the final database;

† Satisfactory indexes; ‡There was a significant difference in precision between the analyzed variables;

§ The mean rate of correct identification of the emotions was 62.5%; //Only images recognized by ≥15 evaluators were included in the final database;

¶ There was no significant difference in precision between the analyzed variables;

# The mean rate of correct identification of the emotions was 66%;

** Only images with ≥60% accuracy were included in the final;

†† Accuracy is presented for each emotion and varied from 44 to 100%;

‡‡ Only images recognized by at least 55% of the evaluators were included in the final database. The mean recognition of the final database was 63%;

§§ The mean recognition rate of the final database varied from 47 to 94%.

### Risk of bias

The studies selected in this review are for the construction of face databases. In this sense, the traditional risk of bias tools used in randomized and nonrandomized studies is not applicable. The task elaborated by the studies must offer valid and interpretable data for the assessment of facial recognition of basic emotions of individuals in certain contexts. Therefore, the quality of the studies included can be observed based on the analyses performed for the reliability and validity of the databases elaborated^18,19^.

### Data analysis

We analyzed the psychometric properties assessed by the studies in the stage for the validation of the stimuli (Table 2)^64,65^. This information is important to assess the quality of the database that was elaborated. Qualitatively, we followed the standards for educational and psychological testing of the American Educational Research Association^20^ and the stages specified in Resolution 09-2018 of the Brazilian Federal Council of Psychology^21^, which regulates the dimensions necessary for the assessment of psychological tests. Consequently, information based on the analysis of the database items and the measures for validity evidence were obtained (Table 2).

In addition, we sought to identify in Table 2 when the psychometric measure assessed by the studies presented satisfactory indexes. For accuracy, as a reference standard we used the consensus among most of the studies on the construction of face databases that include stimuli with recognition rates ≥70%. In some cases, the studies established other rates for recognition, which were indicated as symbols in the table.

Since accuracy is a fundamental indicator for stimuli selection and has been widely used as a quality parameter for construction studies, this variable is included in the table as an indicator of both precision and content-based validity evidence, since it is a precision measure that was used to validate the database content. For agreement among the evaluators, the studies generally use Cohen's or Fleiss’ kappa indexes. Therefore, we used value ≥60% as a reference^22,23^. For internal consistency, we used Cronbach's alpha value >0.70 as a reference^24^.

## RESULTS

### Selection and presentation of the studies

**Figure 1:**
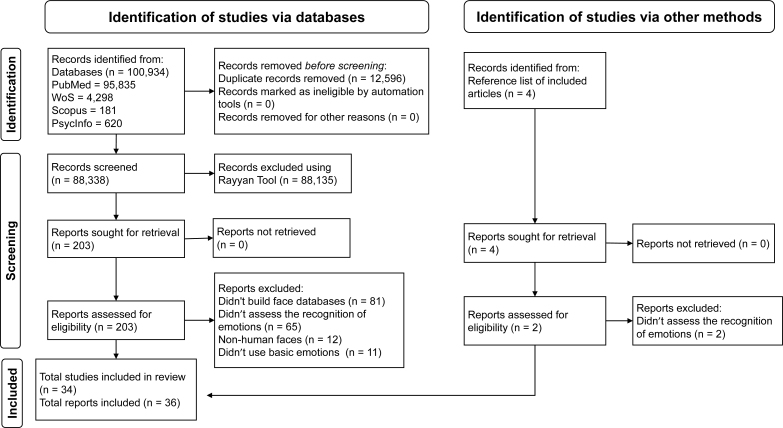
The article selection process according to the PRISMA initiative recommendations^17^.

Figure 1 presents the search and selection process for the 36 articles included in this systematic review^12–17,25–63^.

Table 1 presents the general characteristics of the face databases included and Table 2 presents the methodological characteristics used to create each of them.

### General characteristics of the face databases included

The articles included were published between 1976 and 2020, the majority dating from 2015 and 2017. Of the 36 articles included, 30.56% were carried out in the United States. In relation to the theoretical framework used for the construction of the databases, 75% of the studies were empirically based. In other words, the limitations of the databases already built were the basis for this construction.

Most of the articles (61.1%) elaborated databases made up by six basic emotions (i.e., happiness, sadness, fear, anger, disgust, and surprise), as well as neutral faces. Some databases did not neutral faces, or surprise and disgust. Two databases only included happiness and neutral faces, one database only included happiness, fear, and neutral; and another included only happiness, sadness, anger, and surprise.

In relation to the participants, 41.7% of the studies selected resorted to actors (either amateur or professional) to express the emotions. The mean age of the actors varied from 13.24 to 73.2 years, with four studies including different age groups in their databases. Only five of the studies with actors included different races in their samples, and seven studies included any of the specific race, namely, Caucasian, Japanese, Korean, Polish, Indian, or Chinese. Three studies did not report the actors’ race.

In relation to the other studies, that is, those that present the basic emotions expressed by community-dwelling individuals, inserted in various contexts, presented ages varying from 4 months to 93 years, and five of these studies included volunteers of different ages. Of these, 10 studies included participants of different races and the remaining studies included only one race, namely, Korean, Caucasian, Indian, and Chinese. Three studies did not report the participants’ race. With regard to the presentation of the stimuli, 86.1% of the studies included colored faces in their databases, four studies used black and white faces, and one study included both colored and black and white faces in its database.

Most of the databases included (75%) present static stimuli, four studies are of dynamic stimuli, and five databases have both static and dynamic stimuli. Five studies presented open and closed mouth expressions, and other studies included additional features such as varying intensities and varying angles. The final total stimuli included in the databases varied from 42 to 18,800.

### Methodological characteristics used in the studies

#### Method used to elicit the emotions

The method used to elicit the emotions varied across the studies. In general, more than one method was used in this stage. Predominantly, 44.4% of the studies used specific situations as one of the ways to elicit the intended emotions, such as “Imagine that you have just won the lottery; imagine that you have just lost a loved one.” The studies also used instructions based on the muscle movement of the emotions considering protocols such as the Investigator's Guide for the Facial Action Coding System (FACS), others used a photograph as a model, and others elicited the emotions from photographs and/or videos.

Two studies that built faces with infants and children used an instructional protocol, performed by the parents, to elicit the intended emotions. In one study, the individuals could express the emotion any way they wanted. Three studies elicited emotions in the participants through verbal instructions, such as “Make a happy face” and one study used workshops to teach children how to express basic emotions as well as a Directed Facial Action Task used to guide movement of anatomical landmarks.

#### Recording the stimuli

Most of the studies sought to establish and describe patterns to record the stimuli. For example, the images were photographed against a white background, black, or gray, and the individuals wore black or white garments. In addition, 55.6% of the studies established distractors that should be removed from the volunteers so that the images could be recorded, such as jewelry, accessories, and strong makeup.

#### Validation stage

The number of participants who validated the faces constructed by the studies varied from 4 to 1,362, and most of the participants who validated the stimuli were inserted in a university context. The way to validate the final stimuli in the database varied across the studies. The majority included recognition accuracy as one of the criteria, with images included reaching recognition percentages from >50 to ≥75%. The studies also used other criteria to include the stimuli in the final database, such as agreement among the evaluators.

#### Psychometric properties of the final database

Only one study did not include accuracy as a precision measure. In most of the cases, it was also used to validate the task content and even for item analysis. One study also used the method of halves as a precision measure. In 66.7% of the studies, the stimuli were recognized with ≥70% accuracy.

Test-retest reliability was a variable used to assess task precision in four studies, all presenting satisfactory indexes for this dimension. Regarding the measures of validity evidence, 10 studies used Cohen's kappa or Fleiss’ kappa to validate the task content according to the agreement among the evaluators. All of them presented satisfactory indexes in this dimension. Only one study used Cronbach's alpha to assess internal consistency, also reporting a satisfactory value.

Six studies analyzed the items’ difficulty. Three studies used Item Response Theory (IRT); one study analyzed difficulty according to the intensity and representativeness scores; one study used the Classical Test Theory (CTT); and one study used discrimination.

Two studies presented validity evidence based on the internal structure. One of them used exploratory factor analysis and the other resorted to factor analysis through the two-parameter Bayesian model. In addition, the other study presented validity evidence based on the convergent relationship, presenting a descriptive comparison of the database built with the POFA bank, with satisfactory indexes.

Fourteen (38.9%) studies presented validity evidence based on the relationship with other variables.

## DISCUSSION

The ability to recognize emotional facial expressions can be modulated by variables such as gender, age, and race. In this sense, a number of studies sought to elaborate valid facial expression databases to assess recognition of emotions in specific populations and contexts. However, the methodological heterogeneity among construction studies can make it difficult to create patterns for the construction of these stimuli, regardless of the context and characteristics of who express them. This systematic review sought to gather the studies that built face databases to assess recognition of basic emotions, describing and comparing the methodologies used in its development.

### General characteristics of the face databases included

The way to present the stimuli of an emotion recognition test has already been target of discussions among researchers in the area, since a pioneering study showed that the recognition of static and dynamic facial emotional stimuli involves different neural areas^66^. In this review, most of the studies consist of static stimulus databases. The difference in the recognition of static or dynamic stimuli is still an unanswered discussion, given that some studies report a higher rate of recognition of dynamic stimuli^67,68^ while others point to a minimal or no difference in the recognition of these stimuli^69,70^.

Khosdelazad et al.^71^ investigated the differences in the performance of 3 emotion recognition tests in 84 healthy participants. The results point to a clear difference in the performance of tests with static or dynamic stimuli, with the stimuli that change from a neutral face to the intended emotion (dynamic) being the most difficult to be recognized, given the low performance in the test^71^. However, it is noteworthy that variables such as age and schooling also modulated performance in the tests, highlighting the importance of normative data regardless of the type of stimulus chosen^71^.

Several stimuli databases for facial expressions of emotions were developed in order to be used in specific populations and cultures^72^. Cultural issues must be taken into account when understanding these emotional expressions, as they can exert an influence on their recognition^73^. A study that considered ethnicity as an influencing factor in the performance of emotion recognition tasks and compared this ability to identify emotions between Australian and Chinese individuals verified that people perform worse when classifying emotions that are expressed on faces of another ethnicity^74^. In this sense, the cultural characteristics of the stimulus presented can also modulate performance in the test.

In addition to the difference in the pattern of response when recognizing emotions from another culture, studies showed that there is still a difference in the pattern of intensity recognized, regardless of the race or gender of the stimulus presented^75,76^. This fact happens probably because we manage our emotions according to the our learnings throughout our lives, clearly shaped by the cultural context in which we are inserted^76,77^. Thus, we learned in certain situations to hide or amplify our emotions, consequently affecting how we recognize emotions and highlighting the clear influences of culture on our social and cognitive abilities^76,78^.

Furthermore, when we think about the modulating character of the cultural context in the recognition of emotions, it is important to highlight the impact that socioeconomic status can also have on this ability. In particular, some countries and regions with greater socioeconomic disparities may reflect different patterns of cognitive abilities^79^. For example, a large international study investigated, in 12 countries and 587 participants, the influence of nationality on core social cognition skills^80^.

After controlling the analyses for other modulating variables such as age, sex, and education, the results showed that a variation of 20.76% (95%CI 8.26–35.69) in the test score that evaluated emotion recognition can be attributed to the nationality of the individuals evaluated^80^. These results make us reflect on the cultural disparities that exist in underdeveloped countries and how these aspects can influence the social and cognitive variables, as well as the recognition of emotions discussed here.

In addition, aspects related to the participant's profile can also interfere in task performance. Five studies in this review presented open and closed mouth expressions and other studies included additional features such as varying intensities, gaze directions, and varying angles. These variables can also modulate task performance. Emotions expressed with the mouth open seem to increase the intensity of the emotion perceived by the subject^81,82^. Consequently, incorporating this face variation to the database can be important to assess the emotion experienced by the individual who recognizes the stimuli. In addition, open-mouthed facial expressions seem to draw more the attention of the respondent than closed-mouthed expressions^81^.

Hoffmann et al.^83^ found a correlation between the intensity and accuracy of recognition of an emotion, where higher intensities were associated with greater accuracy in the perception of the face. However, Wingenbach et al.^84^ did not find effects of the intensity level on expression recognition. Despite the controversial results regarding emotion intensity, it can still be an important variable to be taken into account in the construction of databases in order to compare recognition between different degrees of intensities.

The perception of the emotion expressed can also be modulated by the gaze direction of the person expressing it^85^ so that when gaze is directed at the participant, this recognition is greater than when compared to the look avoided^86^. In addition, photographing the expressions from different angles can increase the ecological validity of the database built^38^.

### Methodological characteristics used in the studies

#### Method used to elicit the emotions

An important methodological choice in the studies that elaborate face databases is the way in which the stimuli will be elicited and who is going to express them. Our results show that most of the studies included in this systematic review resort to actors (either amateur or professional) to express the emotions. Such methodological choice can be justified by the fact that people who have experience in acting are able to express more realistic emotions than individuals without any experience^87^. Thus, resorting to actors to act out emotions can be advantageous with regard to bringing the emotions expressed to a more real context.

The literature indicates that there are three different ways to induce emotions, namely:

- Posed emotions;
- Induced emotions; and
- Spontaneous emotions^88,89^.

Posed emotions are those expressed by actors or under specific guidance, tending to be less representative of an emotion expressed in a real context^89^. Induced emotions have a more genuine character than posed emotions, as varied eliciting stimuli are presented to the participant in order to generate the most spontaneous emotion possible^89^. However, it is noteworthy that this way of inducing emotion can also have limitations as to its veracity, since induction is carried out in a context controlled by the researcher^89^. Spontaneous emotions are considered closer to a real-life context. However, due to their observable character, their recording could only be possible when the individuals are not aware that they are being recorded. Thus, any research procedure can bias this spontaneity^89^.

To increase induction effectiveness, the studies use a combination of techniques and procedures to facilitate achievement of the intended emotions. Among the 36 studies analyzed in this review, 44.4% used specific hypothetical situations as one of the ways to elicit the intended emotions, such as “Imagine that you have just won the lottery; imagine that you have just lost a loved one.” Thus, despite induction being generated in a controlled context, using hypothetical everyday situations aims at remedying the limitation of expressions that are not very representative of real life.

#### Recording the stimuli

All construction studies try to capture stimuli following some kind of pattern. Some explore this pattern more in detail and others are more objective. Despite this, the data included in this review indicate that it is important to standardize the clothes worn by the participants and the background they are positioned against during the capture of stimuli.

In addition, most construction studies have established distractors that should be removed prior to image capture, such as jewelry, accessories, and strong makeup. Our hypothesis is that these distractors could direct the attention of those who respond to the task and exert an impact on recognition performance, since attention can be a modulating variable in emotional tasks^90^.

#### Validation stage

The way to validate the stimuli in the databases elaborated varies greatly across the studies. Based on the methods used in the construction, the validation criteria are defined. Accuracy is the most used precision indicator in the development and validation of face databases that assess recognition of emotions^12,13^, which is why it was presented in most of the studies included. Recognition rate ≥70% is the most frequently used. However, the choice of which criterion to adopt at this stage is varied, and it is common to adopt other rates and criteria to validate the database, such as intensity, clarity, and agreement between evaluators.

#### Psychometric properties of the final database

We seek to follow the standards established by Resolution 09-2018 of the Federal Council of Psychology, which regulates the necessary dimensions for the assessment of psychological tests to verify the psychometric qualities of the databases. Although the studies present construction of tasks and not instruments, recognition of emotions is an important skill that allows for interaction in society and can be used to assess social cognition to predict the diagnosis of mental disorders^91^.

The analyses presented by the studies in this stage are also heterogeneous. However, some dimensions presented in the studies become strictly necessary to verify the quality of the database elaborated. With regard to the technical requirements, it is important to evaluate dimensions related to precision and validity evidence of the constructed task^20,21^. It is worth noting that normative data are also important to assess the quality of the task. However, this variable and other important analyses were not included in this review as they are found in articles published separately.

This review showed that the studies that elaborate face databases for the recognition of emotions present heterogeneous methods. However, similarities between the studies allow us to trace important patterns for the development of these stimuli, such as using more than one method to elicit the most spontaneous emotion possible, standardizing the characteristics of the volunteers for capturing the stimuli, validating the database based on preestablished criteria, and presenting data referring to precision and validity evidence. With regard to future directions related to the research methods, greater standardization of the methods for eliciting and validating emotions would make the choice of the type of task to be used in each context more reliable.
